# Frequency and pattern of exercise and depression after two years in older Japanese adults: the JAGES longitudinal study

**DOI:** 10.1038/s41598-018-29053-x

**Published:** 2018-07-25

**Authors:** Satoru Kanamori, Tomoko Takamiya, Shigeru Inoue, Yuko Kai, Taishi Tsuji, Katsunori Kondo

**Affiliations:** 1 Department of Preventive Medicine and Public Health, Tokyo Medical University, Tokyo, Japan; 2 Human Resource Management Department, ITOCHU Techno-Solutions Corporation, Tokyo, Japan; 3 Physical Fitness Research Institute, Meiji Yasuda Life Foundation of Health and Welfare, Tokyo, Japan; 4 Center for Preventive Medical Sciences, Chiba University, Chiba, Japan; 5 Center for Well-being and Society, Nihon Fukushi University, Aichi, Japan; 6 Department of Gerontology and Evaluation Study, Center for Gerontology and Social Science, National Center for Geriatrics and Gerontology, Aichi, Japan

## Abstract

Few have clarified what exercise frequencies and patterns (e.g. alone or with others) are effective for preventing depression in older adults. We examined the relationship between total frequency and/or pattern of exercise and the risk of depression after two years in older Japanese adults. We used a sub-sample of the Japan Gerontological Evaluation Study (JAGES) performed in 2011 and 2013. The sample for analysis was 1,422 adults aged 65 years or older without depression and low physical strength in 2011. All variables were assessed with a questionnaire including the geriatric depression screening scale (GDS-15). Binomial logistic regression analysis was used to examine the relationships between exercise in 2011 and depression in 2013 (0 = non-depression, 1 = depression). The adjusted odds ratio (OR) for later depression was 0.52 (95% confidence intervals: 0.33–0.81) for exercise two or more times a week compared to non-exercisers. The OR for exercisers who exercise with others even a little (Ewo) was 0.53 (0.34–0.84) compared to non-exercisers. Among combinations of frequency and pattern, the OR for Ewo who exercise two or more times a week was 0.40 (0.24–0.68) compared to non-exercisers. Exercising at least twice a week and/or with others may be useful in preventing depression in older adults.

## Introduction

Depression is a common mental disorder. The proportion of the global population with depression in 2015 was estimated to be 4.4%^1^. The total estimated number of people with depression increased by 18.4% between 2005 and 2015^1^. The prevalence of depression was especially high among older adults^1^. Depression in older adults notably lowers their quality of life^2^, increasing their risk for suicide^3,4^, dementia^3^, and functional decline^5^. Therefore, prevention of depression in older adults is an important challenge in public health.

It has been shown that exercise is effective in not only the treatment^6–10^ of depression but also prevention^11^. Exercise has been defined as “a subset of physical activity that is planned, structured, and repetitive and has as a final or an intermediate objective the improvement or maintenance of physical fitness”^12^. There are many different domains of exercise, such as frequency, intensity, type, and duration^13^, which must be considered when attempting to determine its effectiveness in preventing depression^5^. A systematic review of prospective studies showed that any level of physical activity, including low level (e.g. walking less than 150 minutes per week), can prevent later depression^11^. However, more research is needed on dose-response relative to exercise and depression. In addition, the subjects examined in the review ranged widely in age from 11 to 100 years, and findings on older adults specifically are lacking. A study on dose of exercise for adults including older adults suggested that exercising for at least an hour a week can prevent 12% of future cases of depression^14^. Based on a previous study on older adults, consistent exercise with at least 15 min. per session, three times a week of moderate intensity was significantly associated with lower risk of depressive symptoms^15^. This association has not yet been examined in those exercising less frequently than three times a week. If a lower frequency of exercise (such as twice a week) is also effective in prevention of depression, it may be possible to recommend a more age-friendly exercise model.

Exercise can be performed alone or with others^16^. Reviews on exercising with others and various health outcomes (e.g. functional disability or mental health) showed that exercising with others might lead to improved health outcomes through improvement in social relationships, in addition to increased physical activity^16–18^. Randomized controlled trials showed that social relations in exercise programs improved subjective well-being^19^ and loneliness^20^ in older adults. Although depression was not the outcome of these studies, exercising with others may offer not only the biological effects of physical exercise, but may also help prevent depression with the social relations gained through exercise. There are several studies that showed exercising with others improves depression^21,22^. However, to our knowledge, no studies have examined the relationships between depression and exercising with others, also taking the effects of exercising alone into consideration. A cross-sectional study showed a significantly lower prevalence of poor self-rated health among older adults who exercise with others, even sometimes, compared to those who only exercise alone^23^. While a similar relationship may exist for depression as well, such a relationship has not yet been examined. If the relationship with prevention of depression differs when exercising alone versus exercising with others, suggestions for social aspects that are not included in current physical activity guidelines^13^ can be provided with exercise recommendations.

As such, the objective of this study was to examine (1) the relationship between frequency of exercise at baseline and later depression in older Japanese adults and (2) the relationship between exercise patterns at baseline (non-exercisers, exercising alone only, or exercising with others) and later depression, and (3) the relationship between combinations of frequency of exercise and exercise patterns at baseline with later depression. Our hypotheses were that there would be a lower prevalence of depression after two years among those who exercise twice or more per week compared to non-exercisers, that depression would be less prevalent among those exercising alone and those exercising with others two years later compared to non-exercisers, and that there would be the lowest prevalence of depression two years later among those exercising with others twice or more per week among the combinations of exercise.

## Methods

### Study design

A population-based prospective longitudinal study conducted in Japan.

### Participants

The present study was based on a sub-sample of the Japan Gerontological Evaluation Study (JAGES)^24^ performed in 2011 and 2013. The participants were older adults (65 years or older) residing in the urban cities of Kashiwa (area: 115 km^2^, population: 380,963 in 2011), Nagoya (area: 326 km^2^, population: 2,215,062), or Kobe (area: 553 km^2^, population: 1,525,393) who did not have functional disabilities at baseline. Not having functional disabilities was defined as being ineligible for long-term public care insurance benefits^25^. Out of the 46,007 JAGES participants in 2011, one fifth (about 9,200 people) was randomly selected for this study, as the remainder was allocated equally to four other JAGES surveys used in 2011. Participants were mailed self-administered questionnaires in December 2011 as the baseline survey and, if they responded to that survey, again in November 2013 as a follow-up.

Respondents who exhibited depression (GDS-15 ≥ 5) as assessed by the geriatric depression screening scale (GDS-15^26^) during the baseline survey were excluded, as the focus of this study is on the prevention of depression. Those with low physical strength (determined as a part of the Kihon Checklist developed by the Ministry of Health, Labor and Welfare in Japan for assessing frailty status^27,28^) were excluded as they may have had difficulty exercising. Those with missing data on depression, exercising alone, exercising with others, physical strength in the baseline survey, or depression in the follow-up survey were also excluded. Regarding depression and physical strength, failure to respond to even one item resulted in exclusion.

### Measures

#### Depression

Depression was examined in the baseline and follow-up surveys using the geriatric depression screening scale (GDS-15^26^). GDS is an instrument that was developed to assess depressive symptoms and screen for depression in older adults^29^. GDS-15 is one frequently used instrument for depression screening^30^. Based on the previous meta-analysis, the pooled sensitivity and specificity of the GDS-15 were 82.8% and 72.2%, respectively^30^. Scores ranged from 0 to 15 points, where 0 to 4 is considered non-depressed and 5 or above as having depression.

#### Total frequency/pattern of exercise

There are many validated scales among questionnaires concerning physical activity (e.g. International Physical Activity Questionnaire^31^, Saltin–Grimby Physical Activity Level Scale^32^). That said, no validated scales currently exist concerning the frequency of exercising alone or concerning exercise partners or lack thereof. We therefore used two kinds of questions that we created for this purpose. The question, “how often do you exercise alone?” was used to determine the frequency of exercising alone. The question, “how often do you exercise with a relative, friend, or acquaintance?” was used to determine the frequency of exercising with others. For each question, the choices were “hardly ever”, “one to three times a month”, “about once a week”, and “two or more times a week”. Respondents who answered with ‘hardly ever’ to both of these questions were considered non-exercisers. Those who answered ‘two or more times a week’ for either question or ‘about once a week’ for both questions were grouped as ‘exercises two or more times a week’ (2+/wk). All others were grouped as ‘exercises less than twice a week’ (<2/wk).

Regarding patterns of exercise, exercisers who only exercised alone were grouped as ‘only exercises alone’ (Ea-only). All other exercisers were grouped as ‘exercises with others (Ewo).’

In addition, five frequencies/patterns of exercise were classified; non-exercisers, people who only exercised alone less than twice a week (Ea-only (<2/wk)), people who exercised with others less than twice a week (Ewo (<2/wk)), people who only exercised alone two or more times a week (Ea-only (2+/wk)), and people who exercised with others two or more times a week (Ewo (2+/wk)).

#### Covariates

Age (65–69 years, 70–74 years, 75–79 years, or 80 years or more), sex (male or female), living area (Kashiwa, Nagoya, or Kobe), annual equivalized income (less than 2 million yen a year  (low), 2 to 3.99 million yen a year (middle), or 4 million yen or more a year  (high)), educational attainment (less than 9 years, 10–12 years, more than 12 years), household composition (living alone or with others), work status (employed or unemployed), body mass index (BMI: less than 18.5, 18.5 to 24.9, or 25.0 or more), self-reported medical conditions (has an illness/disability or does not have an illness/disability), smoking (non-smoker or smoker), alcohol consumption (drinks alcohol or does not drink alcohol), emotional support (yes or no) were assessed with a questionnaire in the baseline survey.

### Statistical analysis

We performed a binomial logistic regression to calculate the odds ratios (ORs) for depression. The dependent variable was depression (0 = non-depression, 1 = depression) at follow-up. The independent variables were total frequency of exercise, pattern of exercise, or the combined categories of total frequency of exercise and pattern of exercise at baseline. The reference for total frequency of exercise was non-exercisers. Based on previous studies^11,15,23,33^, we adjusted for age, sex, city, annual equivalized income, educational attainment, household composition, work status, BMI, self-reported medical condition, smoking, alcohol consumption, and emotional support at baseline. The reference for pattern of exercise at baseline was non-exercisers. As sensitivity analysis, we performed analysis with people who only exercised alone one to three times a month and people who only exercised with others one to three times a month both classified as non-exercisers.

All variables were set as dummy variables. Based on a previous study^23^, a “missing” category was used in the analysis to account for missing values in response to questions. This method, called the indicator method, is widely used by epidemiologists for handling missing data^34^. The indicator method also has higher precision than the complete-case analysis because a record is included for every individual in the data set.

SPSS 21.0 J was used for statistical analysis with a significance level of 5%.

### Ethics statement

Ethical approval for the study was obtained from the Nihon Fukushi University Ethics Committee (application number: 10–05 and 13–14). This study was performed in accordance with the principles of the Declaration of Helsinki. Informed consent was obtained from all participants.

## Results

**Figure 1:**
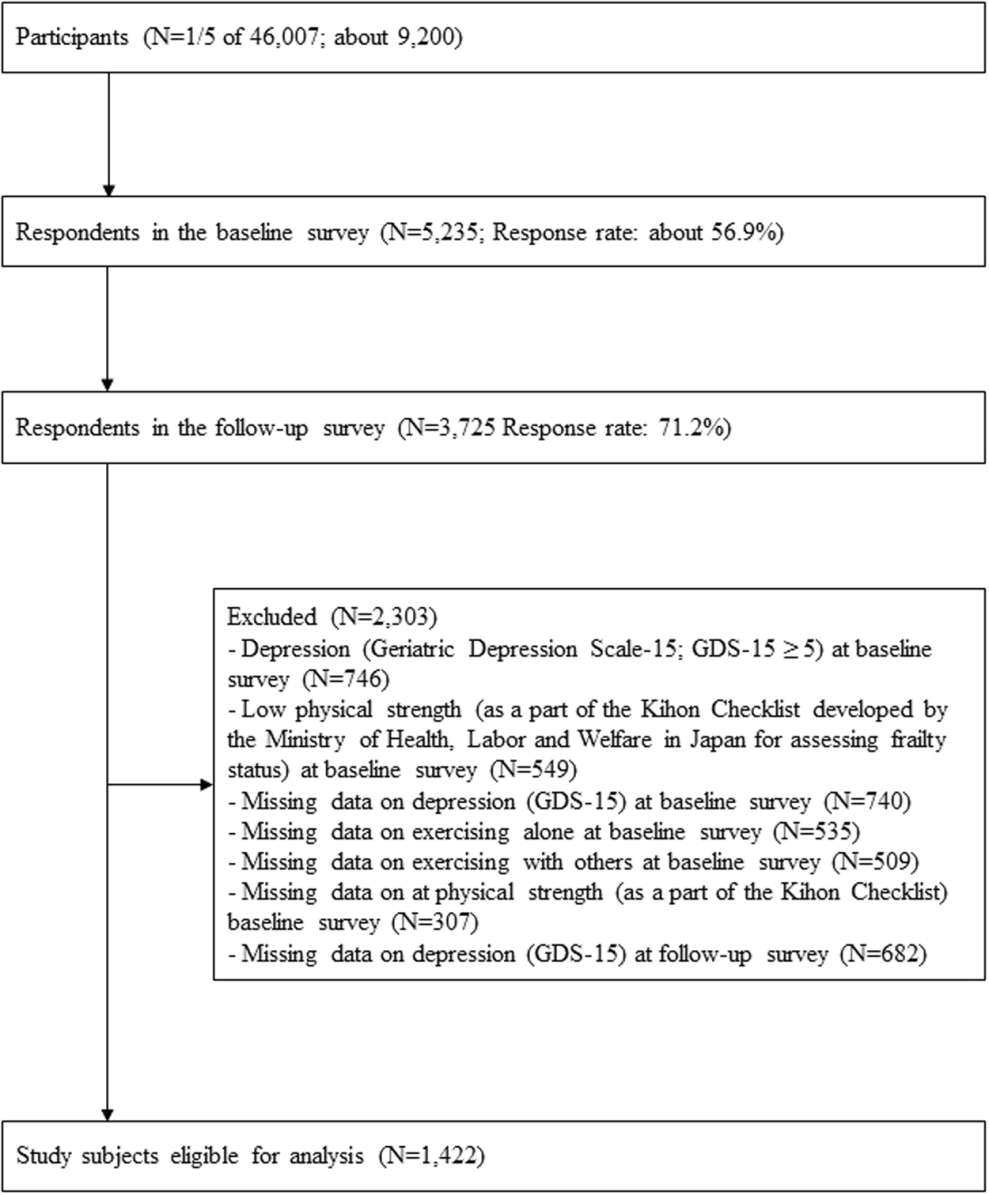
Flowchart of participants.

Among the fifth of the 46,007 participants selected for this study (about 9,200 participants), 5,235 (response rate: about 56.9%) responded to the baseline survey and 3,725 (response rate: 71.2%) to the follow-up survey (Fig. 1). After those who did not meet the required criteria were excluded, the remainder was 1,422 participants who were used in the analysis.

Table 1 shows the characteristics of individuals by frequency and pattern of exercise. The age of subjects was 72.5 ± 4.9 years (mean ± SD). The mean age values for different frequencies and patterns of exercise were as follows: 73.0 ± 5.2 years for non-exercisers; 72.1 ± 4.9 years for Ea-only (<2/wk); 72.1 ± 4.6 years for Ewo (<2/wk); 72.9 ± 5.3 years for Ea-only (2+/wk); and 72.2 ± 4.5 years for Ewo (2+/wk). A total of 348 respondents (24.5%) had a missing value for at least one question. Compared to those with no missing values, there was a greater ratio of those aged 75 or older (39.9% vs 29.0%) and of women (52.0% vs 43.9%) among those with missing values. The most common missing value was for self-reported medical condition that was missing in 8.7% of returned questionnaires.

**Table 1:** Characteristics of individuals according to frequency and patterns of exercise.

		Non-exercisers		Exercisers <2/wk				Exercisers 2+/wk				Total	
				Ea-only		Ewo		Ea-only		Ewo			
		N	%	N	%	N	%	N	%	N	%	N	%
N		364	100.0	145	100.0	163	100.0	270	100.0	480	100.0	1,422	100.0
Exercising alone	Hardly ever	364	100.0	0	0.0	77	47.2	0	0.0	65	13.5	506	35.6
	1–3/mo	0	0.0	63	43.4	59	36.2	0	0.0	11	2.3	133	9.4
	About 1/wk	0	0.0	82	56.6	27	16.6	0	0.0	70	14.6	179	12.6
	2+/wk	0	0.0	0	0.0	0	0.0	270	100.0	334	69.6	604	42.5
Exercising with others	Hardly ever	364	100.0	145	100.0	0	0.0	270	100.0	0	0.0	779	54.8
	1–3/mo	0	0.0	0	0.0	122	74.8	0	0.0	57	11.9	179	12.6
	About 1/wk	0	0.0	0	0.0	41	25.2	0	0.0	111	23.1	152	10.7
	2+/wk	0	0.0	0	0.0	0	0.0	0	0.0	312	65.0	312	21.9
Age (years)	65–69	116	31.9	51	35.2	57	35.0	82	30.4	161	33.5	467	32.8
	70–74	116	31.9	53	36.6	60	36.8	100	37.0	175	36.5	504	35.4
	75–79	85	23.4	27	18.6	29	17.8	49	18.1	108	22.5	298	21.0
	80+	47	12.9	14	9.7	17	10.4	39	14.4	36	7.5	153	10.8
Sex	Male	199	54.7	89	61.4	92	56.4	153	56.7	237	49.4	770	54.1
	Female	165	45.3	56	38.6	71	43.6	117	43.3	243	50.6	652	45.9
Living area	Kashiwa	38	10.4	19	13.1	23	14.1	52	19.3	90	18.8	222	15.6
	Nagoya	211	58.0	75	51.7	81	49.7	123	45.6	220	45.8	710	49.9
	Kobe	115	31.6	51	35.2	59	36.2	95	35.2	170	35.4	490	34.5
Annual equivalized income	Low	149	40.9	54	37.2	53	32.5	97	35.9	157	32.7	510	35.9
	Middle	140	38.5	64	44.1	73	44.8	111	41.1	219	45.6	607	42.7
	High	48	13.2	13	9.0	24	14.7	35	13.0	76	15.8	196	13.8
	Missing	27	7.4	14	9.7	13	8.0	27	10.0	28	5.8	109	7.7
Educational attainment (years)	≤9	112	30.8	29	20.0	34	20.9	56	20.7	107	22.3	338	23.8
	10–12	134	36.8	63	43.4	72	44.2	110	40.7	207	43.1	586	41.2
	13+	108	29.7	51	35.2	54	33.1	100	37.0	159	33.1	472	33.2
	Missing	10	2.7	2	1.4	3	1.8	4	1.5	7	1.5	26	1.8
Household composition	Living alone	50	13.7	16	11.0	19	11.7	45	16.7	52	10.8	182	12.8
	With others	314	86.3	129	89.0	144	88.3	225	83.3	428	89.2	1,240	87.2
Work status	Employed	121	33.2	39	26.9	42	25.8	62	23.0	86	17.9	350	24.6
	Unemployed	225	61.8	103	71.0	110	67.5	194	71.9	379	79.0	1,011	71.1
	Missing	18	4.9	3	2.1	11	6.7	14	5.2	15	3.1	61	4.3
Body mass index (kg/m^2^)	<18.5	30	8.2	6	4.1	8	4.9	16	5.9	33	6.9	93	6.5
	18.5–24.9	251	69.0	113	77.9	124	76.1	189	70.0	368	76.7	1,045	73.5
	25.0+	74	20.3	26	17.9	30	18.4	61	22.6	74	15.4	265	18.6
	Missing	9	2.5	0	0.0	1	0.6	4	1.5	5	1.0	19	1.3
Self-reported medical conditions	No illness/disability	89	24.5	39	26.9	44	27.0	73	27.0	130	27.1	375	26.4
	Present illness/disability	243	66.8	95	65.5	103	63.2	179	66.3	303	63.1	923	64.9
	Missing	32	8.8	11	7.6	16	9.8	18	6.7	47	9.8	124	8.7
Drinking	No	204	56.0	67	46.2	84	51.5	141	52.2	245	51.0	741	52.1
	Yes	143	39.3	73	50.3	70	42.9	122	45.2	214	44.6	622	43.7
	Missing	17	4.7	5	3.4	9	5.5	7	2.6	21	4.4	59	4.1
Smoking	No	298	81.9	122	84.1	131	80.4	239	88.5	415	86.5	1,205	84.7
	Yes	47	12.9	17	11.7	24	14.7	20	7.4	38	7.9	146	10.3
	Missing	19	5.2	6	4.1	8	4.9	11	4.1	27	5.6	71	5.0
Emotional support (recipient)	No	19	5.2	4	2.8	1	0.6	14	5.2	9	1.9	47	3.3
	Yes	334	91.8	137	94.5	159	97.5	253	93.7	462	96.3	1,345	94.6
	Missing	11	3.0	4	2.8	3	1.8	3	1.1	9	1.9	30	2.1

“Ea-only” and “Ewo” means “people who only exercised alone” and “people who exercised with others”, respectively.

Table 2 shows the adjusted ORs for depression according to frequency of exercise and pattern of exercise. In Model 1 that was adjusted for age and sex and Model 2 that was adjusted for the other covariates. For total frequency of exercise, the ORs for exercise two or more times a week was significantly lower than the ORs of non-exercisers (ORs: 0.50 [95% confidence interval, 0.32–0.76] in Model 1, 0.52 [0.33–0.81] in Model 2). As sensitivity analysis, we performed analysis with people who only exercised alone one to three times a month and people who only exercised with others one to three times a month both classified as non-exercisers (Table 3). The ORs for exercise two or more times a week was 0.46 [0.31–0.69] and for exercise less than twice a week was 0.81 [0.45–1.47] in Model 2.

**Table 2:** Odds ratios for depression two years later by total frequency of exercise and pattern of exercise.

	N	Crude		Model 1		Model 2	
		OR	95% CI	OR	95% CI	OR	95% CI
**Total frequency of exercise**							
Non-exercisers	364	ref	—	ref	—	ref	—
<2/wk	308	1.12	0.71–1.76	1.09	0.70–1.72	1.15	0.72–1.85
2+/wk	750	0.50	0.32–0.76	0.50	0.32–0.76	0.52	0.33–0.81
**Pattern of exercise**							
Non-Exercisers	364	ref	—	ref	—	ref	—
Ea-only	415	0.93	0.60–1.43	0.91	0.59–1.40	0.97	0.61–1.52
Ewo	643	0.51	0.33–0.79	0.51	0.33–0.79	0.53	0.34–0.84
**Total frequency of exercise/Pattern of exercise categories**							
Non-Exercisers	364	ref	—	ref	—	ref	—
Ea-only (<2/wk)	145	1.41	0.82–2.41	1.37	0.80–2.34	1.39	0.79–2.42
Ewo (<2/wk)	163	0.88	0.49–1.57	0.86	0.48–1.55	0.94	0.51–1.71
Ea-only (2+/wk)	270	0.69	0.41–1.17	0.68	0.40–1.15	0.74	0.43–1.26
Ewo (2+/wk)	480	0.39	0.23–0.65	0.39	0.24–0.65	0.40	0.24–0.68

Model 1 was adjusted for sex, age.

Model 2 was adjusted for the covariates in Model 1 plus living area, annual equivalized income, educational attainment, household composition, work status, body mass index, self-reported medical conditions, drinking, smoking, emotional support.

**Table 3:** Odds ratios for depression after two years by total frequency of exercise and pattern of exercise: sensitivity analysis with ‘non-exerciser’ defined differently.

	N	Crude		Model 1		Model 2	
		OR	95% CI	OR	95% CI	OR	95% CI
**Total frequency of exercise**							
Non-exercisers*	522	ref	—	ref	—	ref	—
<2/wk	150	0.76	0.43–1.35	0.75	0.42–1.34	0.81	0.45–1.47
2+/wk	750	0.44	0.30–0.65	0.45	0.31–0.66	0.46	0.31–0.69
**Pattern of exercise**							
Non-Exercisers*	522	ref	—	ref	—	ref	—
Ea-only	436	0.71	0.48–1.06	0.70	0.47–1.05	0.77	0.51–1.17
Ewo	464	0.30	0.18–0.50	0.31	0.19–0.51	0.31	0.18–0.52
**Total frequency of exercise/Pattern of exercise categories**							
Non-Exercisers*	522	ref	—	ref	—	ref	—
Ea-only (<2/wk)	109	0.94	0.51–1.73	0.91	0.49–1.68	1.02	0.54–1.93
Ewo (<2/wk)	117	0.28	0.11–0.72	0.30	0.12–0.76	0.30	0.12–0.77
Ea-only (2+/wk)	327	0.64	0.41–1.01	0.64	0.41–1.00	0.69	0.43–1.10
Ewo (2+/wk)	347	0.31	0.18–0.54	0.31	0.18–0.55	0.31	0.17–0.55

^*^While people who did not exercise alone or with others were classified as non-exercisers in Table 2, both people who exercised alone three times or less per month and people who exercised with others three times or less per month were classified as non-exercisers in Table 3.

Model 1 was adjusted for sex, age.

Model 2 was adjusted for the covariates in Model 1 plus living area, annual equivalized income, educational attainment, household composition, work status, body mass index, self-reported medical conditions, drinking, smoking, emotional support.

For pattern of exercise, the ORs for exercisers with others was significantly lower than the ORs of non-exercisers (ORs: 0.51 [0.33–0.79] in Model 1, 0.53 [0.34–0.84] in Model 2). Performing the same sensitivity analysis as above, the ORs for exercisers alone-only was 0.77 [0.51–1.17] and the ORs for exercisers with others was 0.31 [0.18–0.52] in Model 2.

For the combined categories of total frequency of exercise and pattern of exercise, the ORs for Ewo (2+/wk) was significantly lower than the ORs of non-exercisers (ORs: 0.39 [0.24–0.65] in Model 1, 0.40 [0.24–0.68] in Model 2). The ORs of Ea-only (2+/wk) and Ewo (<2/wk) were both lower than 1, but not significantly lower than those of non-exercisers. In the sensitivity analysis, the ORs for Ewo (<2/wk) was significantly lower than the ORs of non-exercisers (ORs: 0.30 [0.12–0.77] in Model 2). However, similar trends were seen for other comparisons: Ea-only (<2/wk) (ORs: 1.02 [0.54–1.93]), Ea-only (2+/wk) (ORs: 0.69 [0.43–1.10]), and Ewo (2+/wk) (ORs: 0.31 [0.17–0.55]).

## Discussion

We examined the relationship between frequency and pattern of exercise and depression after two years among older Japanese adults. Regarding total frequency of exercise, compared to non-exercisers, the risk of depression two years later was significantly lower among those who exercised at least twice a week. This result supports our first hypothesis.

In recent prospective cohort studies, one study found the amount of exercise needed to prevent depression to be at least one hour per week^14^ while another indicated that 15 minutes per session, three times or more per week is required^15^. In the present study, we discovered the new finding that exercise twice a week or more can prevent future depression. With exercise twice a week or more, many of the subjects in the present study may be meeting the volume requirement to prevent depression demonstrated in the aforementioned study of 45 to 60 minutes per week. However, we did not consider duration of exercise in the present study, and cannot make any statements about any aspect other than frequency.

Regarding pattern of exercise, those who exercised with others had a significantly lower risk of depression than non-exercisers. No significant relationship was observed among those who only exercised alone. While these results support part of our second hypothesis, the finding that no relationship was observed for those exercising alone does not support another part of our second hypothesis. Moreover, examinations of the relationships with combinations of frequency and pattern of exercise revealed that the Ewo (2+/wk) group had the lowest risk of depression. In the sensitivity analysis, Ewo (<2/wk) was also as low risk of depression as Ewo (2+/wk). These results generally support our third hypothesis.

Several previous studies have examined the relationship between exercising with others and mental health. In a longitudinal study on Japanese middle-aged adults, those who exercised with others had a significantly lower odds ratio for poor mental health five years later than those who did not exercise^35^. In older Japanese adults who experienced significant damage in the Great East Japan Earthquake, those who increased the frequency of exercise in a group after the disaster had less exacerbation of depressive symptoms^21^. Both indicated that exercising with others lowers the risk of depression. The result of our study was therefore consistent with previous studies. The present study found that, for older Japanese people, those who exercised with others, and especially those who exercised at least twice a week and exercised with others at least some of the time, were able to prevent future depression better than those who did not exercise.

The sensitivity analysis in which we defined non-exercisers as people who exercised alone three times or less per month and people who exercised with others three times or less per month, showed that the ORs for Ewo (<2/wk) were significantly lower than the ORs of non-exercisers. This suggests that the significant relationship between exercising with others and depression later in life depends on exercise frequency, one to three times a month or once a week. However, further studies are needed to determine whether once a week would be a sufficient frequency of exercising with others to reduce the risk of depression.

On the other hand, though Ea-only (2+/wk) presented a lower risk of depression, the relationship was not significant. The odds ratio for Ewo (2+/wk) was 0.40 (0.24–0.68), but it was 0.74 (0.43–1.26) for Ea-only (2+/wk), showing a weaker relationship. Previous studies indicated that, compared with only exercising alone, exercising with others lowers the prevalence of poor self-rated health^23^ and the risk of poor mental health^35^, difficulties in activities of daily living^36^, and functional disability^33^. The results of the present study indicate that exercising with others may have a similar effect on depression.

Compared to only exercising alone, exercising with others may be superior for health outcomes, due to high adherence to exercise, improved psychological factors such as enjoyment and stress buffering, and improved social factors such as social support^16^. Social support may not only be gained when exercising with others, but may also determine exercising with others^37^. In the analyses of the present study, social support was considered a confounder for the relationship between exercise and depression. However, it is possible that the above relationship that was observed may have been a result of the protective effects of new social support and social capital gained through exercise with others against depression. We were unable to consider this point in the present study and it remains a question for future studies.

There are several implications from the results of this study for researchers and practitioners who promote exercise for older adults to prevent depression. Currently, older adults who hardly ever exercise are only recommended to exercise twice a week or more. It is desirable to also recommend that older adults who only exercise alone begin exercising with others at least some of that time or add exercise with others to their routine. In addition, there is a need for education and support for exercise groups and organizations, promotion of exercise at gatherings that are not necessarily intended for exercise (such as senior citizens’ clubs), and the provision of places and opportunities to exercise with others.

There are several strengths to this study. First, to our knowledge, this is the first study to examine the association between frequency and pattern of exercise and later depression in community-dwelling older adults. Second, we used a large population-based longitudinal data set. Third, the sensitivity analyses confirmed the robustness of this study’s findings.

There are some limitations to this study. The first is the possibility of selection bias for analytical subjects. About 40% responded to the surveys at both baseline and follow-up, and only about 16% of study participants were included in the final analysis. It is possible that those who responded to the survey at follow-up were healthier than those who did not respond to the survey at baseline or who only responded to the survey at baseline. Also, as those with low physical strength or missing data were excluded from the analysis and the research was limited to urban areas, the results of this study may not be applicable to anyone other than relatively healthy older adults living in urban areas. Among the study participants, 52.7% responded that their total frequency of exercise was twice a week or more. In the 2016 National Health and Nutrition Survey conducted on a random sample of Japanese citizens, 46.5% of men and 38.0% of women aged 65 and older exercised at least twice a week for 30 minutes a time continuously for one year or longer^38^. While the definition of amount of exercise differs from that of the present study, the results we obtained do not appear to be skewed. It should be noted that there was a very high ratio of individuals who were living with others and of individuals receiving emotional support (about 90% for each). Similar ratios were found in other large-scale studies that we conducted on older Japanese adults^23,39^. This indicates that the population in the present study does not differ greatly from the trends for older adults without functional disabilities in Japan as a whole. The second limitation is that, although we were able to consider frequency of exercise, intensity or duration^13^ and type of exercise^40^ (e.g. walking or golf) that are important in examining the relationship between exercise and health outcomes were not taken into account. For example, concerning exercise duration, a cross-sectional study showed that those who generally exercise with others exercised for a longer amount of time each week than those who generally exercised alone^41^. The present results may simply be reflecting differences in these aspects. The third limitation is that “exercise” and exercising “alone” and “with others” were not clearly defined in the questionnaire. For example, different individuals may interpret exercising alone differently, for example doing light exercise or physical activity such as stretching at home or in the case of going to the gym and exercising next to people they do not know. The fourth limitation is that characteristics of participants at the point of follow-up were not considered. The fifth limitation was that the questionnaires were self-administered, and it is possible that there was some discrepancy between the respondent’s perception and the actual facts. For example, they may have responded more positively due to social desirability bias. The sixth limitation is that, although this was a prospective longitudinal study with data from two points in time, causality cannot be determined. In the future, an interventional study that takes these points into consideration would be useful.

## Conclusion

The results of the present study suggest that exercising two or more times a week and/or exercising with others can lower the risk of depression in older Japanese adults. When promoting exercise to older adults to prevent depression, social aspects should be considered in addition to frequency.
